# Molecular signature of cadmium-mediated neurodevelopmental disorders in prenatal to postnatal stages

**DOI:** 10.17179/excli2025-8322

**Published:** 2025-07-17

**Authors:** Sabiha Sultana Preety, Fahim Rejanur Tasin, Amit Sarder, Debasish Halder, Farjana Yasmin, Chanchal Mandal

**Affiliations:** 1 Biotechnology and Genetic Engineering Discipline, Khulna University, Khulna, Bangladesh; 2 Department of Anatomy & Cell Biology, College of Medicine, University of Florida, Gainesville, Florida, USA; 3 Department of Biology and Biochemistry, College of Natural Sciences and Mathematics, University of Houston, Texas, USA; 4 Department of Gynecology & Obstetrics, Khulna Medical College and Hospital, Khulna, Bangladesh

**Keywords:** Neurodevelopment, Cadmium, Epigenetic alteration, Blood-Brain Barrier, Breast milk

## Abstract

Cadmium can surpass fetal circulation and the blood-brain barrier due to its similar physicochemical properties to those of other divalent metals and causes diverse neuronal disorders. Previous reports have suggested a possible link between epigenetic alterations and neuronal changes in offspring due to cadmium exposure at different developmental stages. Hypermethylation of the glucocorticoid receptor NR3C1 disturbs the development of the hypothalamic-pituitary-adrenal axis, which in turn is responsible for the abnormal cognitive behavior of neonates. In addition, the upregulation of placental miR-509-3p and miR-193-5p expression was found to be the major cause of impaired development of the central nervous system. In this review, the epigenetic mechanism of cadmium-mediated neurotoxicity is described. Moreover, the journey of cadmium from the maternal body to the fetal body through circulation and to the neonatal body through breast milk is also tracked. The vulnerability of developing fetuses to cadmium is an alarming issue. Different types of epigenetic changes, such as DNA methylation, altered miRNA expression and histone modifications, are induced by cadmium and lead to various types of neurodevelopmental disorders. We hope this narrative review will provide distinct knowledge of the transportation of cadmium and its adverse effects on fetal neurodevelopment.

See also the graphical abstract(Fig. 1).

## Abbreviations

Adherens junction - AJ

Alkaline phosphatase - ALP

Arsenic - As

Blood brain barrier - BBB

Body weight - BW

Brain-derived neurotrophic factor - BDNF

Cadmium - Cd

Calcium - Ca

Cd-albumin - Cd-alb

Cd-glutathione - Cd-GSH 

Cd-metallothionein - Cd-MT

Central nervous system - CNS

Congenital heart disease - CHD

Coronin-1a - CORO1A

Dendritic spine - DS

Developmental quotients - DQs

Divalent metal transporter-1 - DMT-1

Folate receptor alpha - FR-α

Full-scale intelligence quotient - FSIQ

Gestational day - GD

Glucose transporters - GLUTs

Glutathione - GSH

Glycoprotein M6A - GPM6A

Hypothalamic-pituitary-adrenal - HPA

Iron - Fe

Lead - Pb

Manganese - Mn

Mercury - Hg

Neural tube defects - NTDs

p21-activated kinase 1 - PAK1

Performance IQ - PIQ

Postnatal day - PNDs

Protocadherin - PCDH

Proton-coupled folate transporter - PCFT

Ras-related C3 botulinum toxin substrate 1 - RAC1 

Reactive oxygen species - ROS

Reduced folate carrier - RFC

Secretory pathway calcium-ATPase - SPCA

Sprague-Dawley - SD

Superoxide dismutase - SOD

Syncytiotrophoblast - STB

Tight junction - TJ

Transferrin - Tf

Transferrin receptor - TfR

Transient receptor potential cation channel subfamily V member 6 - TRPV6

Verbal IQ - VIQ

Zinc - Zn

## 1. Introduction

Neurodevelopment is the process of neurogenesis and the advancement of neurological pathways to increase the ability of the brain to establish memory function and learning processes as well as to expand interpersonal skills. This natural process begins at the embryonic stage and begins with neurogenesis events such as cell differentiation, migration, synaptogenesis, and the organization of synaptic nerves (Miller and Fort, 2018[90]). The complex stages of neurodevelopment in fetuses are largely determined by genetics as well as the maternal socioenvironment and behavior throughout the pregnancy period (Stasenko et al., 2010[130]). Maternal nutrition status, immune action, chemical exposure and daily life practices are regarded as the environment for a developing fetus. The complex composition of these environmental factors, along with genetic make-up, determines the neurodevelopment process (Doi et al., 2022[29]).

Heavy metals are among the greatest threats to the developing fetus, as most metals interfere with fetal organogenesis and neurodevelopment (Jacob et al., 2018[58]). Heavy metals such as lead (Pb), arsenic (As), cadmium (Cd), manganese (Mn), mercury (Hg), etc., have been shown to cause memory deficits and abnormal social behavior when exposed to embryonic or fetal stage (Shah-Kulkarni et al., 2020[128]). Gestational Pb exposure has been shown to affect fetal epigenetics as well as synaptogenesis and neuronal plasticity, resulting in memory and learning deficiencies (Schneider et al., 2013[125]; Tasin et al., 2022[132]). Hg exposure at fetal age is a serious risk factor for cognitive function in the early stages of childhood (Dack et al., 2022[26]). Developmental exposure to As affects the fetal nervous system and has been shown to impact long-term abnormal cognitive function in adolescence (Piao et al., 2023[108]). In addition, exposure to heavy metals as a mixture poses even greater risks to the developing fetus during neurodevelopment and maturation.

Cd is a highly toxic heavy metal that is widely dispersed in the environment and poses a serious risk to human health (Branca et al., 2018[12]; Satarug, 2018[123]). Exposure to Cd has toxic effects on the lung (Zhou et al., 2012[166]), kidney, heart (Cosselman et al., 2015[22]; Fagerberg et al., 2015[36]; Satarug et al., 2017[124]), endocrine system (Al-Saleh et al., 2015[5]), bone (Aoshima, 2012[8]), brain (Yuan et al., 2016[158]), pregnancy (Wang et al., 2018[145]), etc., in adult humans. Owing to the lack of a well-established defense system and tight junctions in the endothelial lining, developing fetuses are even more vulnerable to Cd exposure and can develop various cognitive and physiological malformations in childhood after exposure to low to high doses of Cd. Moreover, childbirth complications were reported due to hormonal imbalances (Stasenko et al., 2010[130]) and endoplasmic reticulum stress (Wang et al., 2012[151]) in the placenta caused by Cd exposure.

**Figure 1:**
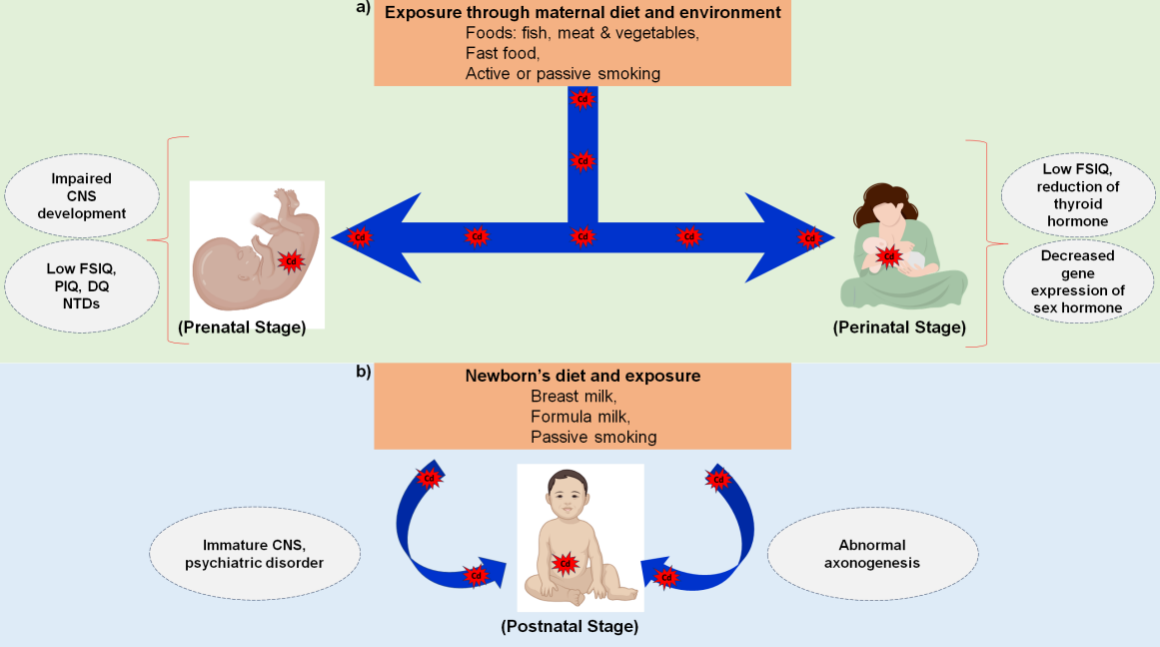
Graphical abstract This is a schematic representation of Cadmium (Cd) exposure and its effect on different stages of development. a) Maternal diet and exposure to Cd affect the prenatal and perinatal stages. The disorders found in the fetus during the prenatal stage are low full-scale intelligence quotient (FSIQ), performance IQ (PIQ), developmental quotients (DQ), neural tube defects (NTDs) and impaired central nervous system (CNS) development and during the perinatal stage are low FSIQ, a reduction in thyroid hormones, and decreased gene expression of sex hormones in the brain. b) On the other hand, the newborn's diet and exposure to Cd affect the postnatal stage, which can induce abnormalities such as immature CNS, abnormal axonogenesis, and psychiatric disorders.

The mechanism of Cd-mediated toxicity has been extensively explored in the last few decades. Interactions between Cd and important cellular sites result in the production of reactive oxygen species (ROS) and cause DNA damage, lipid peroxidation, depletion of antioxidants and alterations in calcium (Ca) homeostasis (Méndez-Armenta and Ríos, 2007[87]). Recent studies have shed light on the underlying mechanisms of Cd-induced toxicity in developing fetuses at different stages of development in the mother's womb. Cd also interferes with the fetal epigenome and dysregulates different gene functions important for neuronal cell differentiation, synaptogenesis and proper closure of the neural tube (Zhang et al., 2016[159]). In this review, we have summarized the sources and metabolism of Cd as well as neurodevelopmental complications associated with epigenetic modifications caused by Cd during *in utero* exposure. A summarized figure is presented in Figure 1(Fig. 1) (graphical abstract).

## 2. Metabolism and Transportation of Cd in the Maternal Body

### 2.1 Sources of Cd in the maternal body

**Figure 2:**
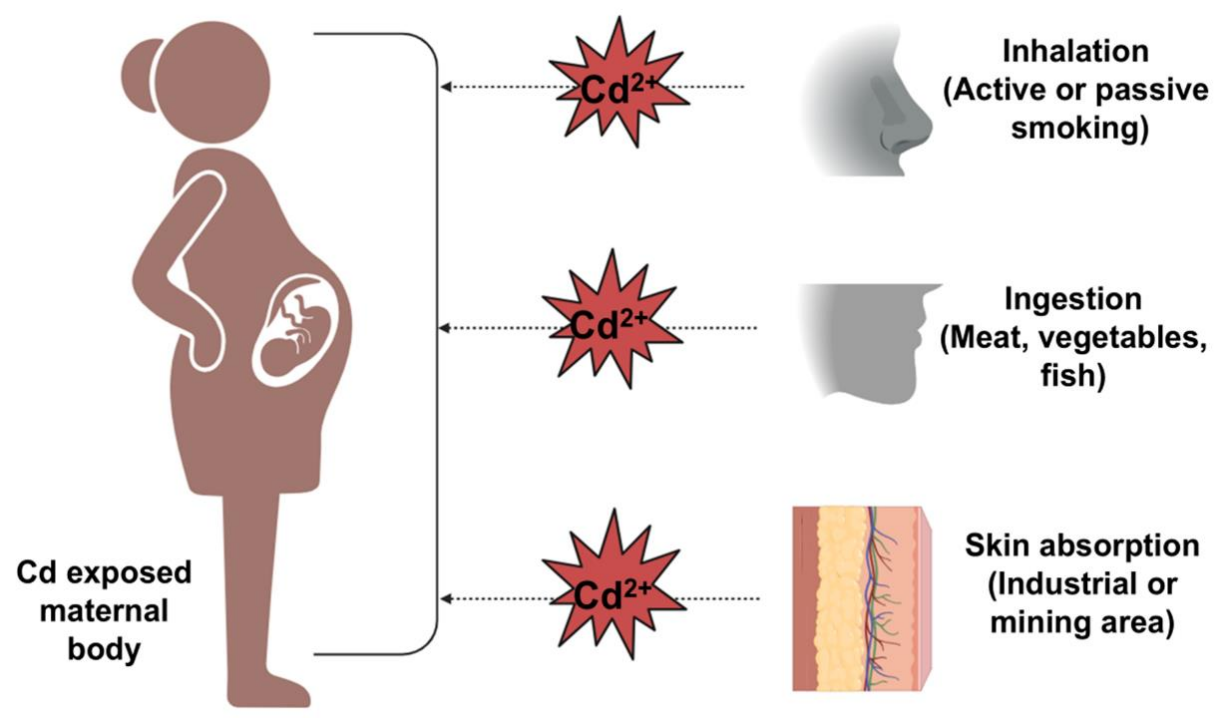
Sources of Cd exposure in the maternal body. There are numerous sources of Cd that lead to exposure to the maternal body. In addition, all these sources have different pathways for entry into the body, such as inhalation, ingestion and skin absorption. Tobacco is one of the major causes of Cd exposure and is inhaled via active or passive smoking. On the other hand, foods enriched with Cd, such as meat, fish, and vegetables, enter through the ingestion pathway. Another less recognized route of Cd exposure is skin absorption. This happens when pregnant women come in contact with Cd-contaminated areas such as industrial or mining sites.

Cd is found in the nature as a compound in association with different elements e.g., oxygen (CdO), chlorine (CdCl_2_), sulfur (CdSO_4_·xH_2_O). Cadmium oxide (CdO) is the most common form found in the environment. A higher transformation rate of Cd to the plants from the soil was reported previously than other heavy metals like Pb and Hg (Satarug, 2018[123]; Zhang and Reynolds, 2019[160]). Humans are exposed to dietary Cd mainly through vegetables and grains (Fagerberg et al., 2015[36]). Consumption of liver and kidney of animal origin represent the major sources of Cd accumulation in human body (Mahurpawar, 2015[85]). Heavy metal exposure can be high in specific areas either geographically or through human actions. For example, vegetable items from South Asian regions, such as potato, onion, bitter gourd, spinach, chili, and tomato, have been shown to accumulate more Cd than the safe limits in different published studies (Nawab et al., 2018[99]; Salhotra and Verma, 2017). The Cd concentration has also been reported to be alarming in fish (Ali et al., 2020[4]; Arulkumar et al., 2017[11]) and meat items in different regions (González-Weller et al., 2006[45]; Jamil et al., 2015[61]). Tobacco is another source of Cd for not only people who actively smoke but also those who are indirectly exposed to smoking. Even people who are rarely exposed to smoke may experience a twofold increase in the lifetime Cd burden (Morrow, 2001[97]). In addition to being ingested and inhaled, Cd can also be exposed to the human body via skin absorption, as our skin is the easiest means of contact with this metal. Cd-rich environments, such as industrial or mining areas, can be sources of exposure through our skins (Wang J et al., 2022[148]). The different sources of Cd via which the maternal body is exposed to this metal are illustrated in Figure 2(Fig. 2).

### 2.2 Path of Cd from the diet to the bloodstream

Cd is generally taken up by humans as Cd-glutathione (Cd-GSH) or Cd-metallothionein (Cd-MT) or in complex with phytochelatins by vegetables or animal body parts. Cd seems to be absorbed in the intestine by enterocytes, mainly in the duodenum and proximal jejunum (Sabolić et al., 2010[117]). Physiological processes in females, such as menstruation and pregnancy, induce a low iron (Fe) status in the body and subsequently increase dietary Cd uptake through gastrointestinal absorption (Fagerberg et al., 2012[37]). The ability of Cd to share physicochemical features such as Ca and zinc (Zn) helps its absorption by competing with these divalent metallic ions (Martelli et al., 2006[86]).

After intake, Cd binds with intestinal metallothionein (MT), and then, Cd-MT is reinternalized, possibly by endocytosis, followed by degradation in lysosomes (Sabolić et al., 2010[117]). When dissociated from complex forms, Cd may exist in the blood in the form of Cd^2+^ or Cd-albumin (Cd-alb). 

The Cd-alb complex enters the liver via endocytosis and dissociates into Cd^2+ ^and albumin via the lysosome.

Cd^2+^ can cross the sinusoidal membrane via divalent metal transporter-1 (DMT-1), Zrt-Irt-like protein-8 or Ca-channels, induce the nucleus to produce MT and can be stored in hepatocytes as a nontoxic Cd-MT complex (Fagerberg et al., 2012[37]). Although some Cd-MT is released into the blood and others into the bile, chronic exposure to low doses of Cd (a few micromoles in the murine model) has been shown to deplete the GSH level by vacuolizing Cd-GSH (Mezynska and Brzóska, 2018[88]). However, short term exposure to a higher dose of Cd might result an elevated Cd^2+^ in the bloodstream which induce ROS in hepatocytes (Sabolić et al., 2010[117]).

The portion of Cd that is not received by hepatocytes is taken up by different tissues (such as the kidney and ovary) after it reaches the systemic circulation (El Muayed et al., 2012[32]; Varga et al., 1993[142]). DMT-1 and, to some extent, transient receptor potential cation channel subfamily V member 6 (TRPV6) play key roles in transporting Cd^2+^ from the systemic circulation to the fetal circulation by crossing the placenta (Dwivedi et al., 2013[31]; Somsuan et al., 2019[129]; Storm et al., 2016[131]).

**Figure 3:**
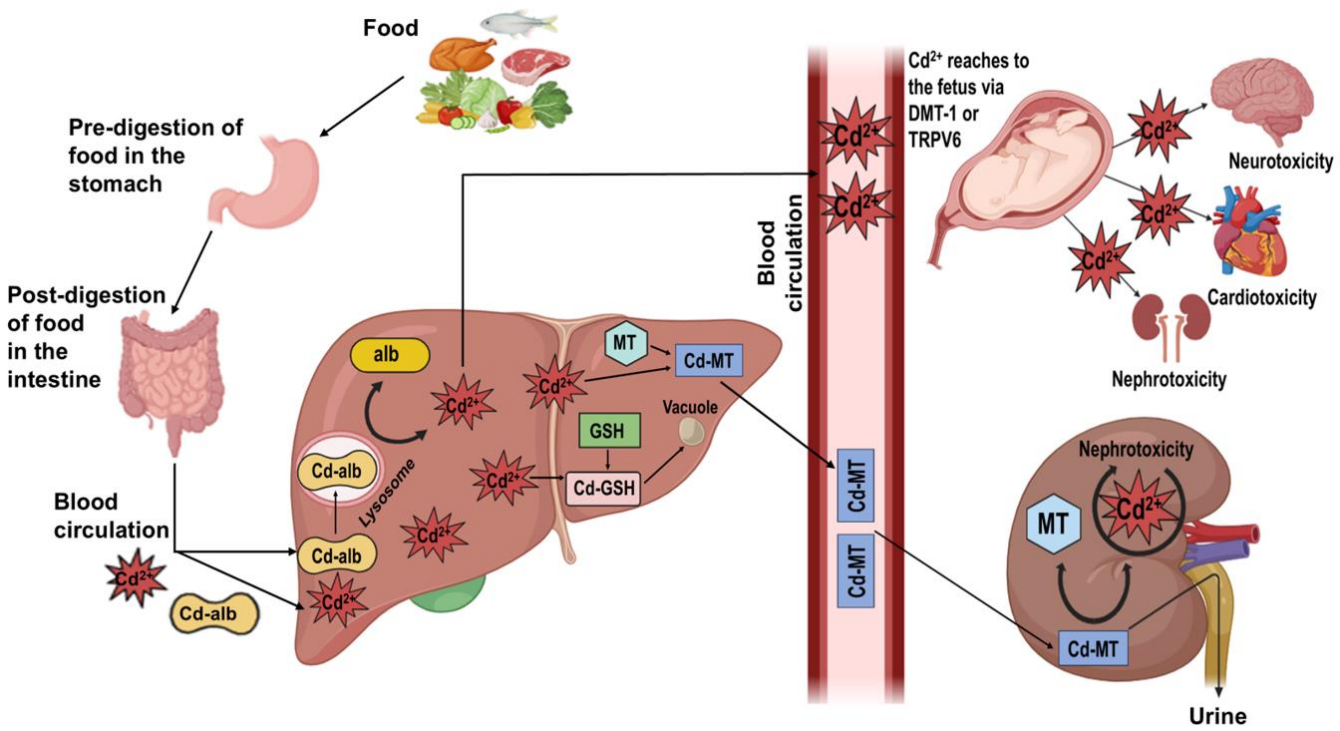
Cd transportation from the diet to the bloodstream. After oral consumption, foods are predigested in the stomach, followed by postdigestion in the intestine. Both free Cd^2+^ and the Cd-alb complex, which originates from digested foods, enter the liver via the blood circulation. Cd-alb is then degraded by lysosomes into Cd^2+ ^and alb. Both the dissociated and the free Cd^2+^ then attach to MT and GSH and form Cd-MT and Cd-GSH, respectively. The vacuolization of Cd-GSH then occurs. Some Cd^2+ ^ions reach the fetal circulation via transporters such as DMT-1 and TRPV6, where they are responsible for triggering toxicity in different organs, such as the brain, heart, and kidney. From the liver, the Cd-MT complex is redistributed in the kidney, where it is either excreted in the urine or broken down into Cd^2+^ and MT. This dissociated Cd^2+^^,^ which is redeposited in the kidney, is responsible for nephrotoxicity.

The redistribution of Cd from the liver occurs in the kidney as Cd-MT (Jarup et al., 1983[62]). This Cd-MT is then excreted in the urine (Fagerberg et al., 2012[37]) or can be redeposited in the kidney if the Cd level reaches the critical concentration (150 µg/g), causing several defects (Fagerberg et al., 2015[36]). The pathway of Cd transportation from the diet state to the bloodstream state is presented in Figure 3(Fig. 3).

### 2.3 Transportation of Cd to the developing fetus

A highly specialized fetal organ, placenta that starts to form approximately 5 days after conception (Turco and Moffett, 2019[140]). The placenta is responsible for the passage of nutrients like Zn and Fe through maternal circulation into the fetal circulation. It also synthesizes important pregnancy hormones such as estrogen, progesterone and other necessarypeptides after the first trimester (Somsuan et al., 2019[129]; Wong et al., 2015[153]). A reduction in the biosynthesis of progesterone from the placenta was noted due to gestational exposure to Cd (Miceli et al., 2005[89]).

**Figure 4:**
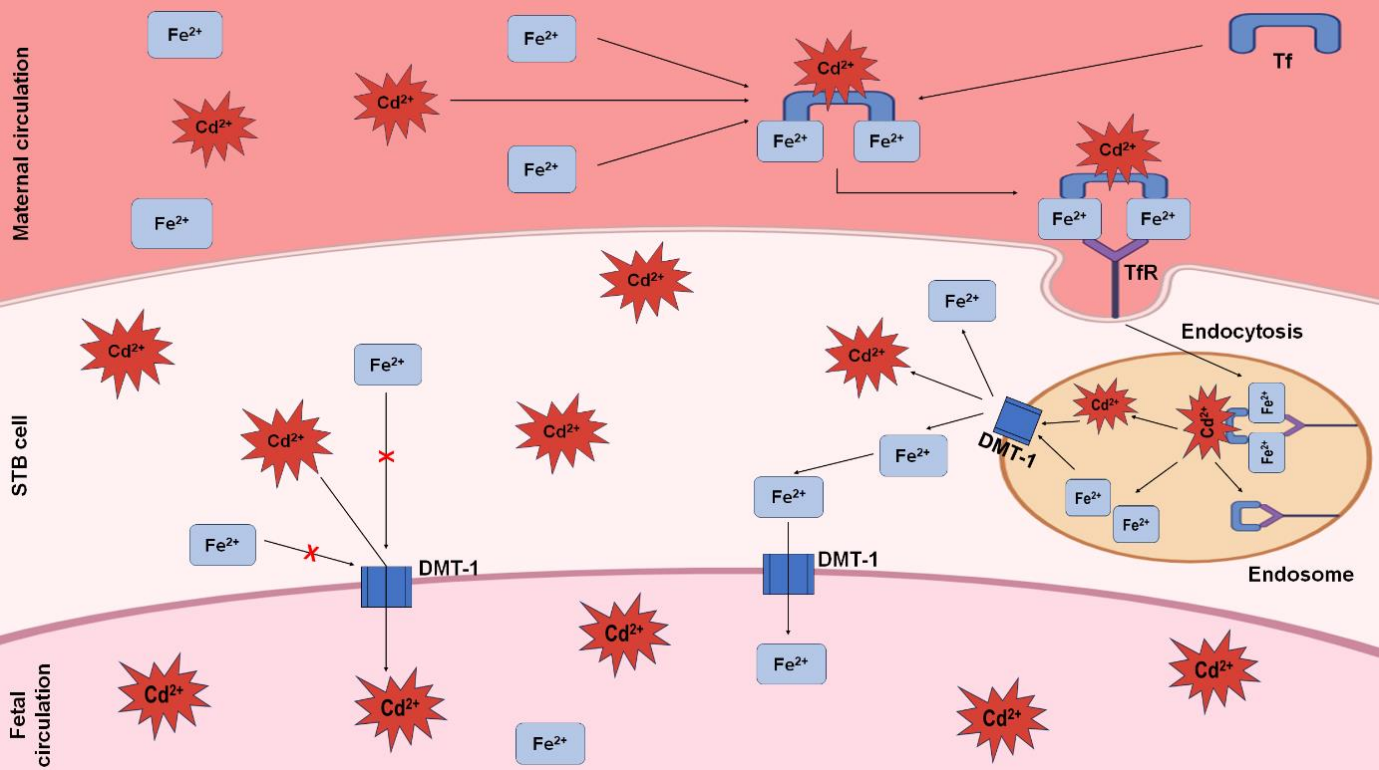
Transportation of Cd from maternal circulation to fetal circulation. Free Cd^2+ ^binds with Fe^2+^ and Tf, forming a complex that then binds with TfR. This conjugate is then endocytosed into the STB cells. After that, DMT-1, located on the endosome's membrane, exports Fe^2+^ and Cd^2+^ into the cytoplasm of the STB. A portion of Cd^2+^ then enters the fetal circulation by competing with Fe^2+^. DMT-1 acts here as a carrier to transport Cd^2+^ and Fe^2+^ into the fetal circulation.

Pregnancy-related exposure to Cd may have an impact on the fetus's vascular system, which results in a decrease in placental blood flow and in the region where the mother and fetus exchange gases and nutrients. This raises the possibility of intrauterine growth retardation (Larsen et al., 2002[75]). As a divalent cation, Cd^2+^ can be transported into fetal circulation in a similar way to that of Fe. In maternal circulation, Cd^2+^ and Fe^2+^ attach to transferrin (Tf) and then contact placental syncytiotrophoblast (STB) cells with the help of transferrin receptor (TfR) (Somsuan et al., 2019[129]). Through endocytosis, they then move toward the endosome. DMT-1, which is located on the surface of the endosomal membrane, predominantly transports Fe^2+^ and Cd^2+^ into the STB cytoplasm. A portion of the Cd^2+^ conjugates with MT, and some Fe^2+ ^is stored in the cytoplasm by forming a bond with ferritin. When Cd^2+^ crosses the threshold level in STB cells, it competes with Fe^2+^ to reach fetal circulation with the help of DMT-1 (Somsuan et al., 2019[129]). The transportation of Cd from maternal circulation to fetal circulation is shown in Figure 4(Fig. 4).

To preserve brain homeostasis and safeguard the central nervous system (CNS), the blood‒brain barrier (BBB) is formed by brain capillaries in vertebrates and functions as a selective barrier (Tjakra et al., 2020[135]). In fetuses, the formation of the BBB starts at the 8^th^ week of gestation (Virgintino et al., 2000[144]). The transportation of nutrients and waste material as well as the development of adherens junctions (AJs) and tight junctions (TJs) are performed by the endothelial capillary cells of the BBB. These two junctions are responsible for limiting the paracellular diffusion of water-soluble particles (Abbott et al., 2010[1]).

Owing to the sophisticated structure of mammalian capillaries in brain tissues, it is not easy to display the toxic nature of Cd in the BBB. However, several studies have been performed on animal models to visualize this trait. A study revealed that when the embryos of zebrafish were treated with CdCl_2_, the cerebral hemorrhage rate increased, which indicates BBB dysfunction (Zhang T et al., 2021[162]). Zonula occludens-1 (ZO-1), claudin-5 and occludin are three important proteins that play key roles in maintaining the integrity of junctions. When two groups of chickens were fed a Cd-rich diet at different concentrations (70 mg/kg CdCl_2_ and 140 mg/kg CdCl_2_), the first group prevented the perturbation of Cd toxicity by upregulating ZO-1 and Occludin slightly, whereas the other group failed to do so. This occurred because of the decrease of these proteins in the presence of a high dose of Cd. Consequently, this leads to damage to these two junctions, TJs and AJs, which are responsible for developing defects in the BBB. This experiment confirmed that Cd can surpass the BBB of the fetus (Li et al., 2023[77]). 

**Figure 5:**
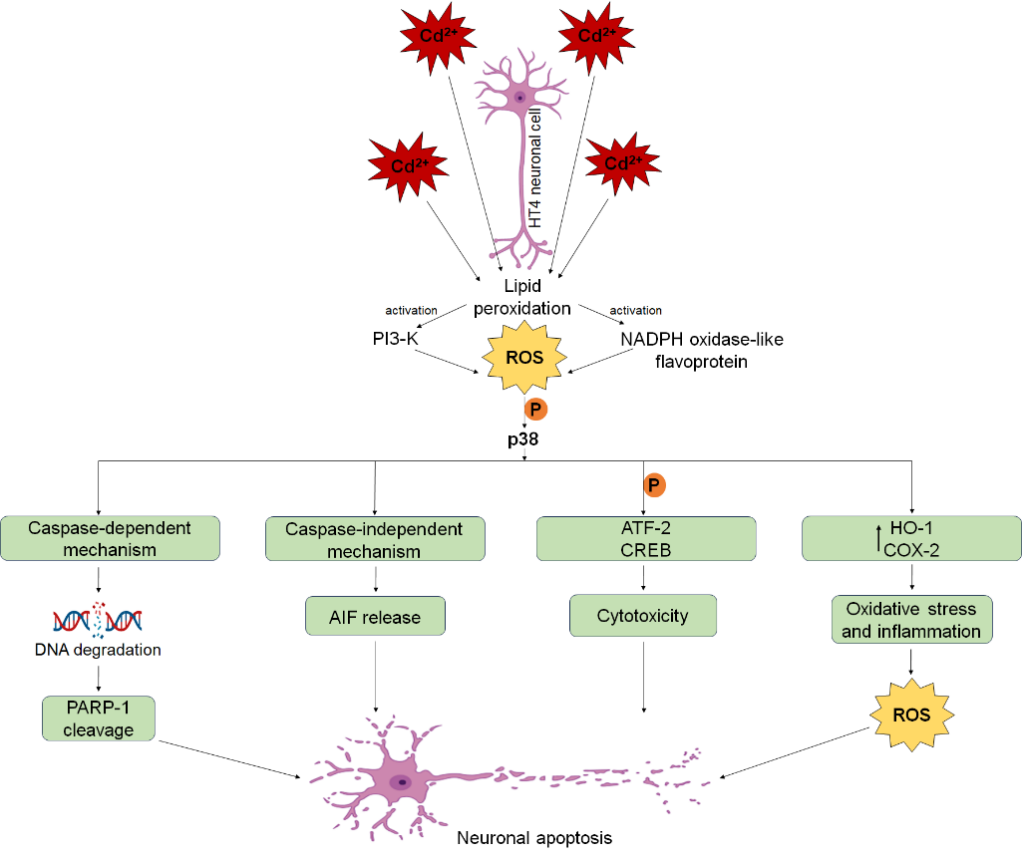
Neuronal cell death via intracellular signaling. The schematic presentation illustrates the Cd-induced molecular events in HT4 neuronal cells leading to apoptosis. Following the lipid peroxidation phase, PI3-K and NADPH oxidase-like flavoprotein are activated initially. This activation contributes to increased reactive oxygen species (ROS) production which in turn initiates a signalling cascade. Here, p38 activation is depicted as a central event that mediates oxidative damage, ultimately leading to neuronal apoptosis. In this figure, phosphorylation steps are indicated by P and arrows represent activation steps. The entire cascade highlights the oxidative stress-related pathway implicated in neurodegenerative processes.

Besides, studies have demonstrated that Cd induces the elevation of ROS generation which leads to BBB disruption, tubulin modifications, and disturbances in synaptic transmission. Cd-induced oxidative stress has been strongly linked to the development and progression of Parkinson's disease and Alzheimer's disease, contributing to neuronal damage and dysfunction (Okuda et al., 1997[102]; Panayi et al., 2002[103]). This ROS generation plays a key role in activating the mitogen-activated protein kinase (MAPK) pathway, contributing to neuronal apoptosis (Pearson et al., 2001[106]). The MAPK family, including extracellular signal-regulated protein kinases (Erk1/2), c-Jun N-terminal kinase (JNK), and p38 MAPK, regulates various cellular processes (Rockwell et al., 2004[113]). These MAPKs become activated during Cd-induced ROS generation (Chen, Luo, et al., 2008[19]). A proposed model for the signaling pathway involved in Cd²⁺-induced oxidative stress in HT4 neuronal cells suggests that ROS production, driven by PI3-K and a NADPH oxidase-like flavoprotein, initiates the activation of stress kinases (JNK and p38 MAPK) and their downstream transcription factors (c-Jun, ATF-2, and CREB). In this pathway, p38 MAPK (but not JNK) acts as a key signaling molecule responsible for the upregulation of COX-2 and HO-1, phosphorylation of ATF-2 and CREB, and the activation of caspase-dependent PARP cleavage and caspase-independent mechanisms, which ultimately contribute to neuronal cell death as shown in Figure 5(Fig. 5) (Rockwell et al., 2004[113]).

### 2.4 Cd exposure during perinatal and postnatal periods

The perinatal and postnatal stages are the two consecutive stages of a newborn. According to the WHO, the perinatal period begins just after the 22^nd^ week of gestation and ends on the 7^th^ day after birth (Cioni et al., 2011[21]). On the other hand, the postnatal period starts just after the birth of a newborn and lasts till 6^th^ to 8^th^ week of life (Lopez-Gonzalez and Kopparapu, 2024[83]). As the perinatal stage consists of both the gestational period and the post-life period, Cd exposure occurs in this case first via cord blood (Li et al., 2019[76]; Zhang M et al., 2021[161]) and secondarily via breastmilk (Samiee et al., 2019[120]). On the other hand, the potential sources of Cd exposure during the postnatal period are thought to be breastmilk (Dabeka et al., 2011[25]), formula milk (Winiarska-Mieczan, 2014[152]) and tobacco smoke (Sanders et al., 2015[121]). Therefore, in both cases, breastmilk has been found to be one of the most common crucial factors.

**Figure 6:**
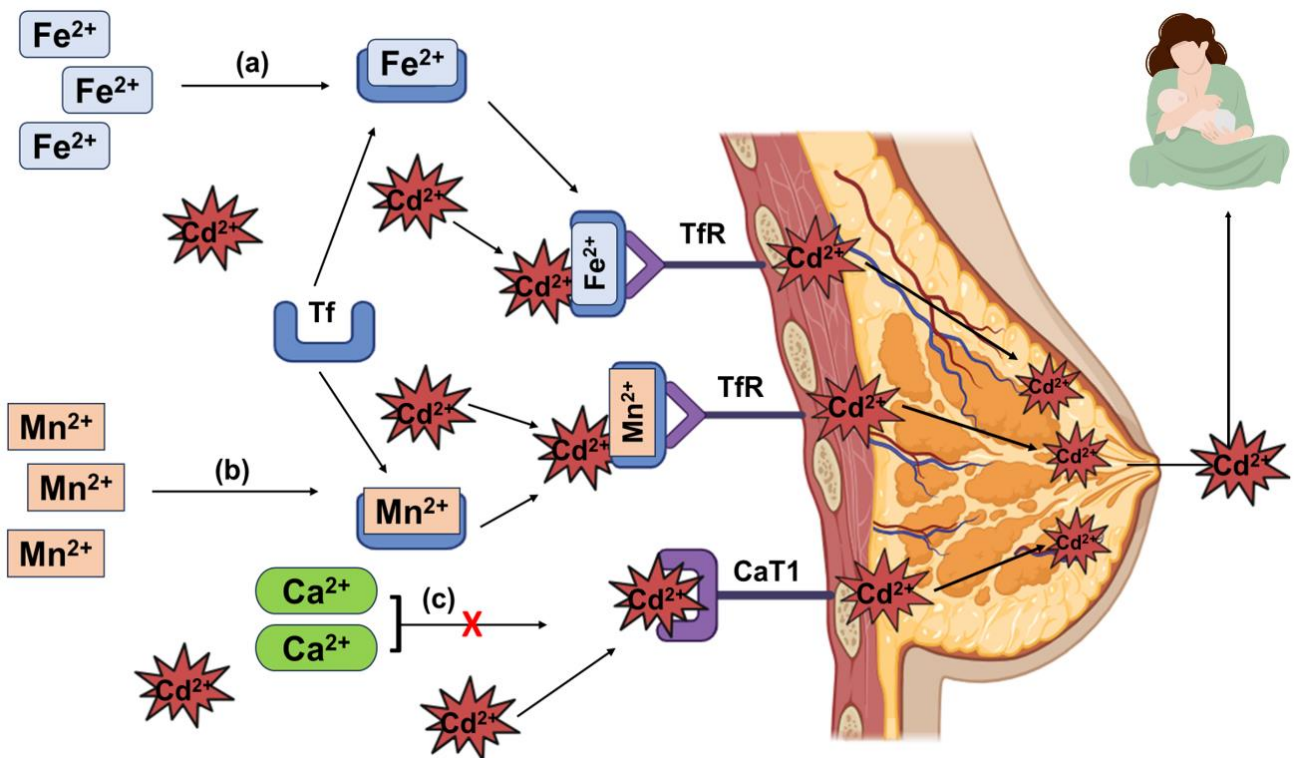
Mechanism of Cd exposure via breastmilk. Cd^2+^ is transported into the mammary gland via the Fe^2+^ and Mn^2+^ pathways. When the Fe^2+^ and Mn^2+ ^of blood plasma bind with transferrin (Tf) separately [(a), (b)], Cd^2+^ is attached to Tf without replacing Fe^2+^ and Mn^2+^. These complexes are subsequently transferred onto the mammary gland with the help of the transferrin receptor (TfR). Thus, TfRs mediate the transportation of Cd^2+^ into the mammary gland. On the other hand, Cd^2+ ^prevents Ca^2+^ from binding with CaT1, a Ca transporter, and instead binds itself with CaT1. In this way, CaT1 facilitates the transportation of Cd^2+^ in the mammary gland. Finally, Cd^2+ ^is transferred to neonates via breastmilk.

The mammary glands that produce breastmilk, separate maternal blood and breastmilk, similar to the placenta (Rossipal, 2000[116]). Although essential nutrients are transported via this gland, it cannot prevent all toxic elements like Cd from entering the breastmilk (Cousins et al., 2006[23]; Nakamura, Ohba, and Ohta, 2012[98]). Postnatal exposure to Cd via breastmilk was confirmed by research on rat offspring, where Cd was found in the stomachs of offspring at post-natal day (PND) 2, PND6 and PND14 before weaning (Jacquillet et al., 2007[60]). Although breastmilk exposure to Cd has been confirmed, the mechanism of this exposure is still unclear. Owing to its similar physical and ionic properties, Cd mimics the transportation pathway of some divalent ions, such as Fe, Mn, and Ca (Bridges and Zalups, 2005[13]). Cellular TfRs are responsible for the transportation of Fe from blood plasma to mammary epithelial cells when they are bound to Tf. (Lönnerdal, 2007[82]). Cd binds with TfR along with Fe when there is a decreased amount of Fe^2+^ and when TfR is upregulated. This affects the transfer of Cd into breastmilk via the Fe transportation pathway (Harris and Madsen, 1988[50]). On the other hand, a positive association was found between Cd^2+^ and Mn^2+,^ where Mn first binds with Tf. Then, it is received by the TfR and transported to the mammary gland via DMT1 (Gunshin et al., 1997[47]). Another experiment revealed that the expression of the milk protein β-casein was reduced as the Ca concentration in the mammary gland was reduced due to the high dose of Cd (Öhrvik et al., 2006[101]). Even a low dose of Cd can inhibit the transportation of Ca via Ca transporters such as CaT1 (Peng et al., 2000[107]). Cd^2+^ also disturbs the function of the mammary gland by downregulating the expression of secretory pathway calcium-ATPase (SPCA). SPCA transports Ca^2+^ into the Golgi apparatus of the mammary gland, which facilitates milk protein phosphorylation (Faddy et al., 2008[35]; Öhrvik et al., 2011[100]). The mechanism of Cd exposure via breastmilk is depicted in Figure 6(Fig. 6).

## 3. Cd Mediated Neurodvelopmental Disorders in Different Stages of Development

### 3.1 Neurodevelopmental disorders in the prenatal stage

The prenatal period is the timeframe before the birth of infants, during which the developing embryo or fetus remains distinctly sensitive to maternal behavior and exposure. In humans, the prenatal stage is categorized as 0-2 weeks as the germinal stage, 3-8 weeks as the embryonic stage, and 9 weeks until birth as the fetal stage. Among these, the fetal stage is critical for migration, maturation, and networking of neurons, and maternal exposure to teratogens poses a high risk to these processes (Ptak and Petronis, 2010[109]). One of the earliest studies showed that prenatal exposure to Cd at gestational day 20 (GD20) created vacuolated endothelial cells in the rat fetal brain, indicating the susceptibility of the CNS to this toxic heavy metal (Rohrer et al., 1978[114]). Gestational Cd exposure at the prenatal stage has been shown to disturb motor coordination and the sensorimotor reflex, causing a delay in the development of the righting reflex and cliff aversion at 0.6 mg/kg Cd exposure in pregnant Wister rats (GD7to GD15) (Minetti and Reale, 2006[92]). A South Asian study assessing urinary Cd levels during pregnancy revealed an inverse association not only with the full-scale intellectual quotient (FSIQ) but also with verbal IQ (VIQ) and performance IQ (PIQ) at the age of 5 years (Kippler, Tofail, Hamadani, et al., 2012[73]). Exencephaly was identified in the fetuses of C57 and SWV mice at GD18, as pregnant mothers were injected with 4 mg/kg CdCl_2_ on GD8 (Robinson et al., 2009[111]). One of the earliest studies on rats exposed to 3.5 mg/kg Cd from GD5 to 15 revealed a significant dose-dependent decrease in vertical and horizontal explorative action among pups due to altered bioelectric and higher-order functions of the CNS (Dési et al., 1998[28]).

In humans, prenatal Cd exposure was shown to be inversely associated with children's adaptive and social skills. Maternal health and behavior also play a role in infants' neurodevelopment due to the high level of susceptibility to toxicants during the gestational period. A negative correlation was observed between prenatal Cd exposure and neurodevelopment in offspring born to diabetic and smoking mothers (Ma et al., 2021[84]). The study considered 300 mothers in 2010 and 2011 in Shandong, China, and measured the infants' developmental quotients (DQs) based on gross and fine motor, adaptivity, linguistic and social traits at one year of age and the level of brain-derived neurotrophic factor (BDNF), an important regulator of synaptic plasticity. They reported that a 10-fold increase in Cd in mothers' blood is associated with 5.7- and 4.31-point decreases in DQ and cord serum BDNF levels, respectively (Wang Y et al., 2016[149]). Studies performed in other countries have also revealed a correlation between maternal Cd levels and impaired cognitive development (Kippler et al., 2016[69]) and lower PIQ (Jeong et al., 2015[63]) among offspring.

### 3.2 Neurodevelopmental disorders in the perinatal stage

The perinatal period starts at later stages of gestation and ends after the first week of childbirth in humans. Synaptic action during the perinatal period is also recognized as a decisive period in neurodevelopment since the synaptic maturation and apoptosis of inactive neurons occur predominantly at perinatal age (Cioni et al., 2011[21]).

A separate cohort revealed an inverse association between the Cd level in cord blood and the FSIQ of offspring at the age of 4.5 years (Tian et al., 2009[134]). Studies have been conducted on rodents and murine models exposed to Cd at late gestational and early-life nursing ages, which exhibit abnormal behavioral patterns. A murine model study with perinatal Cd exposure revealed that perinatal Cd exposure downregulates the expression of specific hormones in the brain at low doses (Ishitobi et al., 2007[57]). An important regulator of early brain development is thyroid hormone. It has been observed that perinatal Cd exposure in mice caused a significant decrease in serum thyroxine levels in MT-I/II-null mice (Mori et al., 2006[96]). A study conducted in 2004 in Taiwan revealed that high cord blood Cd levels (mean 0.31 µg/L) were associated with significant decreases in head circumference and decreases in height, weight, and head size up to 3 years of age (Lin et al., 2011[79]).

### 3.3 Neurodevelopmental disorders in the postnatal stage

Treatment with Cd or 'Cd and Pb' significantly decreased the levels of the CNS maturating enzyme alkaline phosphatase (ALP) on PND0 in Cd‒Pb-co-exposed pups. Another enzyme important for synaptic function, acetylcholinesterase (AChE), has been shown to be decreased at PND21 in the Cd-exposed group (Antonio et al., 2003[7]). Low explorative skills among the pups were identified in an experiment performed on Wister rats. The mother rats were fed with 7 mg/kg Cd from gestational day (GD) 5 to GD 15 and 4 lactation weeks, and it was assumed that the abnormalities occurred due to altered bioelectric and higher-order functions of the CNS (Dési et al., 1998[28]).

An overall presentation of neuro-related disorders caused by different stages of Cd exposure and their molecular events are presented in Table 1(Tab. 1) (References in Table 1: Antonio et al., 2003[7]; Dési et al., 1998[28]; Feng et al., 2019[39]; Ishitobi et al., 2007[57]; Jeong et al., 2015[63]; Kippler et al., 2012[72], 2016[69]; Lin et al., 2011[79]; Ma et al., 2021[84]; Minetti & Reale, 2006[92]; Mori et al., 2006[96]; Rohrer et al., 1978[114]; Tehrani et al., 2023[133]; Tian et al., 2009[134]; Wang et al., 2016[146]; Zhang et al., 2016[159]; Zhou et al., 2020[165]).

## 4. Potent Obstruction of Neural Tube Closure and Neuronal Growth by Gestational Cd Exposure

Neural tube defects (NTDs) are reported as the most common developmental disorders of neonates. Cd exposure during Neurulation interrupts the closure of neural tubes and reportedly causes exencephaly in different experimental animal models (Ferm, 1971[40]; Fernandez, 2003[41]; Paniagua-Castro et al., 2007[104]). Unlike Pb, most Cd cannot pass through the placental barrier and is mostly stored as a neutral Cd‒MT complex, which raises the question of how this metal causes neural tube deformities in newborns. The placenta, as a critical mediator of fetal development, regulates numerous nutritional passages and generates neurotransmitters involved in fetal neurodevelopment. A growing body of evidence suggests that most abnormalities in the cognitive behavior of children are associated with high placental and cord blood Cd levels (Hudson et al., 2021[53]; Liu et al., 2021[80]; Tung et al., 2022[139]). Therefore, injuries or altered expression of placental genes hampering their functionality increase the risk of decreased nutrient transport and subsequent occurrence of neuronal disorders in newborns.

The formation of neural tube occurs at the embryonic day 15 in human which in turn develops into the spinal cord and the brain. This neurulation process depends on the availability of folate (Czeizel et al., 2013[24]). Folate compounds help to synthesize nucleotides (Rochtus et al., 2015[112]) and regenerate methionine from toxic homocysteine (Czeizel et al., 2013[24]; Saini et al., 2016[119]). Human body intake folate from cereals, vegetables, legumes and different types of fruits. Methionine is involved in the closure of neural tube by distributing tubulin and actin in neuroepithelial cells (Imbard et al., 2013[55]). A maternal intake of 0.4 to 0.8 mg of folate can reduce the risk of NTDs as suggested by scientists from USA (Bibbins-Domingo et al., 2017[44]).

Therefore, low folate intake resulting in decreased folate concentrations in fetal circulation increases the risk of NTDs, such as anencephaly, exencephaly and encephalomeningocele, leading to life-threatening outcomes. The transfer of folate across the placenta is mediated by several placental proteins, such as folate receptor alpha (FR-α), reduced folate carrier (RFC) and proton-coupled folate transporter (PCFT) (Zhao et al., 2011[164]). A particular mouse model study revealed a significant decrease in the PCFT level in Cd-treated mothers on GD-8, whereas the other two FR-αs and the RFC remained unaffected. Consequently, lowered placental weight and a lack of folate transfer were noted, and the offspring developed anencephaly, exencephaly and encephalomeningocele at different doses of exposure (Zhang GB et al., 2016[159]).

Critical neurodevelopmental steps are regulated by different evolutionarily conserved homologous or orthologous genes studied in different study models (Tropepe and Sive, 2003[138]). Embryonic brain development in the zebrafish model is analogous to that in vertebrates, and a particular study revealed that a high Cd burden on embryos results in the development of abnormal neural progenitor cells and mid-hindbrain boundaries. A previous study revealed altered neurogenesis, neuronal differentiation and axonogenesis in Cd-treated embryos (Chow et al., 2008[20]). In a later stage, neurite formation relies largely on actin-rich, finger-like projections called filopodia that act as precursors of dendritic spines (DSs). The morphological plasticity of DS is considered a critical factor for learning and memory development in newborns (Sekino et al., 2007[127]). The formation of filopodia in the CNS is directly mediated by Coronin-1a (CORO1A) and its downstream signaling molecules, Ras-related C3 botulinum toxin substrate 1 (RAC1) and p21-activated kinase 1 (PAK1), which induce glycoprotein M6A (GPM6A) localization (Alvarez Juliá et al., 2016[6]). Experimentally, 5 mg/kg body weight (BW) CdCl_2_ treatment of pregnant rats resulted in significant downregulation of offspring CORO1A-RAC1-PAK1-GPM6A signaling, consequently inhibiting normal synapse formation and neuronal growth at early postnatal days (Feng et al., 2019[39]). Reduced PAK1 activation may increase the risk of Alzheimer's disease by affecting the hippocampus (Zhao et al., 2006[163])

On the other hand, it was identified that pregnancy-related exposure of Cd disrupts estrogen balance, leading to cognitive impairment in offspring (Liu et al., 2022[80]). This exposure reduces the expression of estrogen synthesis enzymes: CYP17A1, CYP19, CYP11A1, and StAR, consequently lowering estrogen levels in both the placenta and fetal brain (Xiong et al., 2021[154]; Zhu et al., 2021[167]). This phenomenon hampers the brain development by decreasing the activation of the E2/ERα signaling pathway. Reduced estrogen leads to lower levels of BDNF, PSD95, Synapsin-1, and NR2A in the fetal brain, impairing synaptic plasticity and cognitive function in adulthood. These changes primarily occur in the placenta and fetal brain, contributing to long-term neurological deficits like Alzheimer's disease (Liu et al., 2022[80]; Zhao et al., 2006[163]). Another crucial component for the formation of neuronal filopodia is the neuronal membrane protein GPM6A, which was shown to be targeted by the same study (Feng et al., 2019[39]).

**Figure 7:**
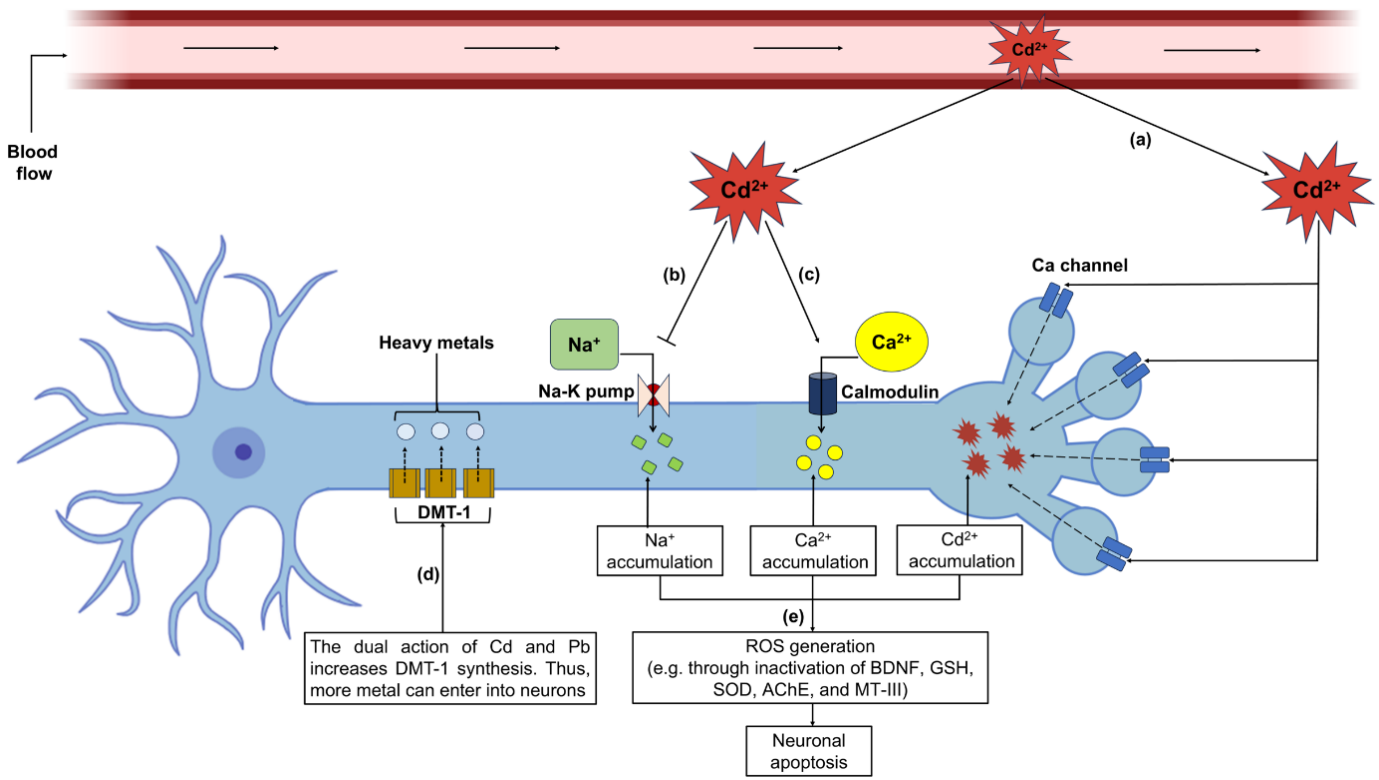
Cd-induced death of fetal neurons. (a) Cd^2+^ in fetal blood flow crosses the BBB and enters neurons via Ca channels. (b) Cd^2+^ inactivates the Na-K pump, resulting in the accumulation of Na^+^ in neurons. (c) Ca^2+^ accumulates in neurons via calmodulin activation. (d) The dual action of Cd and Pb increases DMT-1 synthesis; thus, more heavy metals can enter neurons and increase the ROS level. (e) Intracellular accumulation of Na^+^, Ca^2+^ and Cd^2+^ leads to ROS generation and neuronal apoptosis.

The human brain remains separated from the circulation by the BBB. However, owing to less maturity, the permeability of the fetal BBB is greater than that of the adult brain; thus, a serious threat remains after heavy metal exposure at the gestational stage (Jacobo-Estrada et al., 2017[59]). Cd is known to cross the BBB of the developing fetus which in turn is deposited in the fetal brain (Jacobo-Estrada et al., 2017[59]). In general, Cd^2+^ mimics Ca^2+^ and enters neurons through the Ca channel, activates calmodulin expression (Xu et al., 2011[155]), disrupts Ca homeostasis, and subsequently reduces intracellular GSH, catalase, MT-III, BDNF, and superoxide dismutase (SOD) activity, resulting in increased intracellular ROS levels (Karri et al., 2016[66]; Xu et al., 2011[155]). In an earlier experimental rodent study, *in utero* Cd exposure reduced Na^+^ and K^+^-TPase activity and AChE activity in the brains of pups, resulting in lower weights than those of the controls (Gupta et al., 1991[48]). Disturbances in Na^+^/K^+^-ATPase pumps may result in increased Na^+^ accumulation and subsequent deposition of ROS molecules (Figure 7(Fig. 7)) (Xu et al., 2011[155]). High ROS content can activate the Akt/mTOR pathway along with caspase-dependent and caspase-independent mitochondrial signaling, which can lead to neuronal apoptosis (Yuan et al., 2016[158]), neurotransmitter balance and synaptic function (Rai et al., 2010[110]; Saghazadeh and Rezaei, 2017[118]), increasing the risk of autism-like syndrome among newborns. In response to coexposure to Cd, As and Pb reduce glial fibrillary acidic protein expression and increase the apoptosis of astrocytes synergistically in the developing rat brain (Rai et al., 2010[110]). An *in utero* binary mixture of Pb and Cd induces DMT-1 synthesis in the fetus's brain, enhancing Fe^2+^ transportation into the neurons. Increased metal content might increase ROS levels and subsequently impair fetal cognitive development (Karri et al., 2016[66]).

## 5. Epigenetic Mechanisms Underlying Neurodevelopmental Disorders

### 5.1 DNA methylation

By studying epigenetics, we understand how gene expression is regulated in response to environmental factors without altering the sequence of genes (Abdi et al., 2018[3]; Iridoy Zulet et al., 2017[56]). Among all other epigenetic modifications, DNA methylation is the most well-known mechanism that explains the emergence of certain diseases (Iridoy Zulet et al., 2017[56]). DNAmethylation has been used as a biomarker to identify the epigenetic alterations that lead to different neurological diseases (Jin and Liu, 2018[65]). Cd exposure has been shown to have detrimental epigenetic effects on different human tissues that can cause abnormalities in fetal circulation and neurodevelopment. The effects of epigenetic changes caused by Cd exposure on placental and fetal development are well known and generally involve DNA methylation (Castillo et al., 2012[16]; Kippler et al., 2013[70]; Sanders et al., 2014[122]; Vaiserman and Lushchak, 2021[141]; Vidal et al., 2015[143]).

Protocadherins like PCDHAC1 expression in the placenta may inversely be affected by maternal Cd exposure, potentially making fetal development more susceptible to epigenetic changes. The Pcdh-αC1 gene family encodes a wide range of transmembrane proteins that are expressed in distinct patterns across individual neurons in the vertebrates. Lower DNA methylation in the Pcdh-αC1's promoter and 5'- region appears to increase vulnerability to Cd-related repression of PCDH, which is linked to the impairment of CNS (Kawaguchi et al., 2008[67]). It was also reported that Cd-mediated suppression of Pcdh-αC1 gene in placenta caused preeclampsia in expecting mothers. Reduced expression of this gene can result in neuronal impairment and weight loss in fetuses (Everson et al., 2016[34]). The placenta is the most vital intermediary organ during pregnancy and is involved in the organization and maturation of the hypothalamic-pituitary-adrenal (HPA) axis as well as cortisol homeostasis in the fetus. The actions of the placenta in this pathway rely on the glucocorticoid receptor gene (NR3C1) and its downstream signaling (Seckl, 1997[126]). A growing body of evidence indicates that alterations in the DNA methylation pattern of the NR3C1 gene's promoter can result in neurobehavioral complications in neonates at an early age (Bromer et al., 2013[14]; Monk et al., 2016[94]). According to multivariate regression analysis, prenatal Cd exposure alone or in combination with other heavy metals, such as As, Pb, Mn, Hg and Zn, leads to increased NR3C1 methylation in the placenta. These findings suggest that fetal HPA axis development is disrupted after NR3C1 hypermethylation, which contributes to the risk of reduced neuromuscular activity and abnormal cognitive behavior (Appleton et al., 2017[9]; Bromer et al., 2013[14]). 

### 5.2 Alteration in miRNA expression

miRNAs are among the leading posttranscriptional regulators. The development and functionality of the brain are highly dependent on the proper expression of miRNAs (Wang Z et al., 2022[150]). miRNAs regulate the major number of mRNAs that are expressed in the CNS (Tonacci et al., 2019[137]). The adverse effects of Cd during pregnancy include regulation of the expression of miRNAs like miR-26a. It was found that Cd leads to the inhibition of miR-26a which is responsible for the increased expression of SMAD1 gene, resulting in preeclamptic placenta (Brooks et al., 2016[15]). However, the impact of Cd exposure on the altered expression of miRNAs are responsible for neurodevelopmental disorders, is still under investigation.

A very recent population-based miRNA-seq study revealed an association between altered placental miRNA expression and gestational Cd exposure. They concluded that prenatal Cd exposure markedly increased placental miR-509-3p and miR-193b-5p expression, which corresponds to abnormal placental function and CNS development in fetuses. They predicted 44 target genes (ADAM10, CRK, OTX2 etc.) for miR-509-3p and 47 target genes (ABL2, CIC, SYN6 etc.) for miR-193b-5p, that are involved in CNS development and maturation. The dysregulation of these genes results in atypical neurobehavioral performance of the newborn (Tehrani et al., 2023[133]). Another finding suggests that prenatal Cd and polychlorinated biphenyls exposure alters miRNA expression in the placenta, which may impact fetal development. According to this study, Cd exposure affects miR-1537, a gene linked to high-risk neuroblastoma (Fieuw et al., 2011[42]; Q. Li et al., 2015[78]).

### 5.3 Histone modification

Another epigenetic mechanism through which Cd generates neurotoxicity is histone modification (Ijomone et al., 2020[54]). Among the 16 diverse types of histone modifications, the acetylation and deacetylation of histones are the major factors involved in the emergence of different types of diseases (Chen et al., 2006[18]; Ijomone et al., 2020[54]). In the case of Cd exposure in the fetus, neurotoxicity generated by histone modification seems very rare. In addition to other histone modifications, an interaction between histone deacetylation and Cd-induced neurotoxicity in the fetus has been identified. Histone deacetylases (HDACs) are enzymes that regulate chromatin structure via the deacetylation of histone proteins (Montgomery et al., 2009[95]). HDAC2 (a class of HDACs) has a negative effect on the plasticity of synapses and the formation of memory (Guan et al., 2009[46]). Therefore, psychiatric disorders may occur due to the abnormal upregulation of this enzyme (Datta et al., 2018[27]). When SD rats and their pups were fed water containing Pb acetate and CdCl_2_ (150 ppm and 5 ppm, respectively), an increase in HDAC2 due to the effect of Cd was detected in the hippocampal tissues of the pups (Zhou et al., 2020[165]). A negative relation was found between HDAC2 and some synaptic genes (Bdnf promoter I/II, Egr1, Fos, Cpg15) where these genes' expression was downregulated and was found to be responsible for hampering synaptic plasticity and formation of memory (Guan et al., 2009[46]).

**Figure 8:**
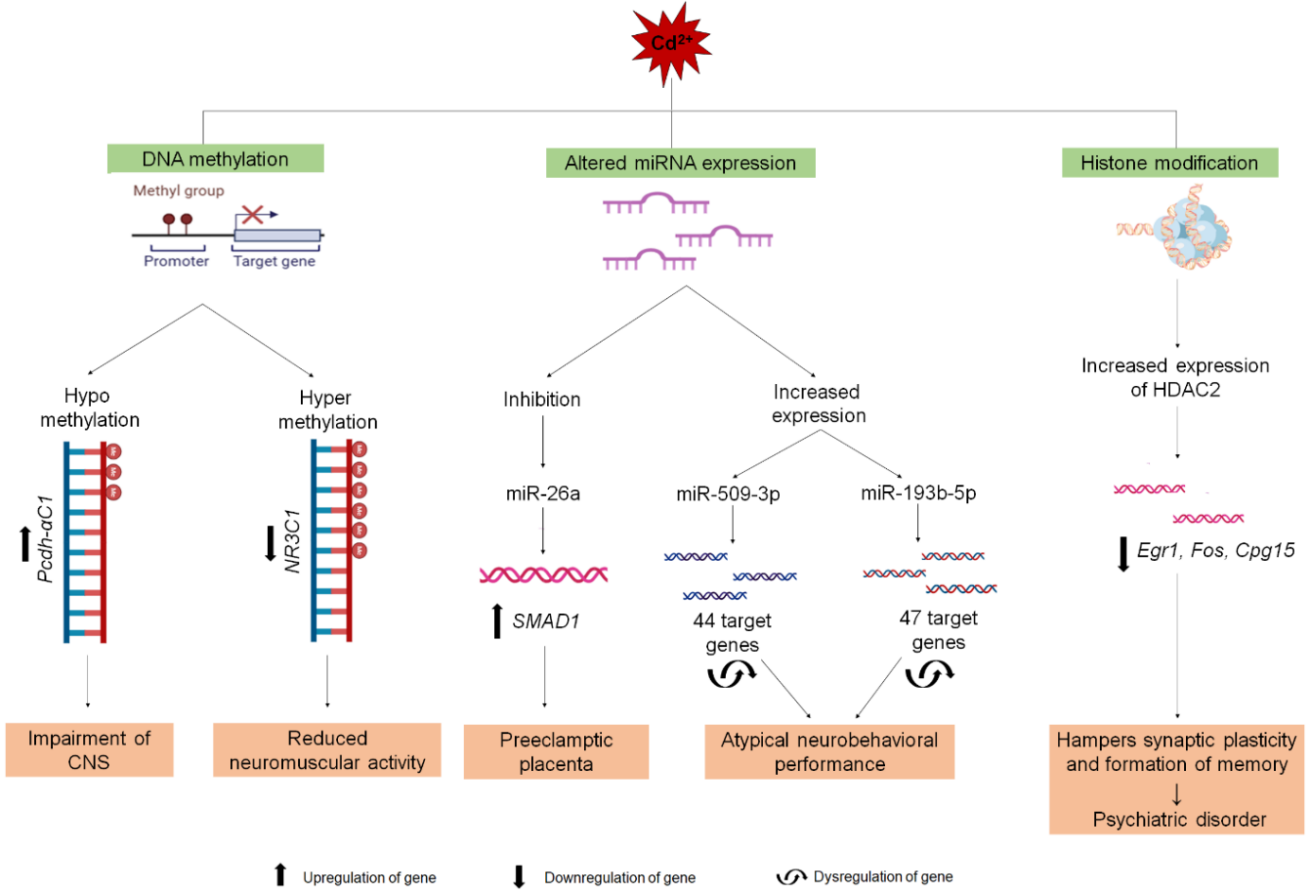
Cd-induced epigenetic modification. DNA Methylation: Cd²⁺ alters DNA methylation patterns affecting genes. Due to the hypermethylation of NR3C1 and hypomethylation of PCDHAC1, cognitive and neuronal impairment are noticed, respectively in the offspring. Altered miRNA Expression: Cd²⁺ exposure modifies the expression of miRNAs (miR-26a, miR-509-3p and miR-193-5p), leading to preeclamptic placenta and atypical neurobehavioral performance. Histone Modification: Cd²⁺ influences histone modifications via increased expression of HDAC2 levels, which is associated with psychiatric disorders. Here, three different types of arrows denote different patterns of gene regulation (upregulation, downregulation and dysregulation) and the names of the genes are written in italic.

A particular study on the strain-specific effects of Cd exposure on neuronal development reported that C57BL/6 mouse pups are more vulnerable than the SWV strain to Cd exposure. Prenatal exposure to Cd markedly upregulated p53 signalling pathway as well as its downstream mediators, Ccng1 and Pmaip1 in C57BL/6 mice. The upregulation of these two genes is associated with NTD. Moreover, several other genes important in CNS development, such as Nr2f1, Nr2f2, Elavl4, Zic1, Neurog1, En2 and Metrn, are reportedly downregulated in C57BL/6 mouse embryos compared with SWV mice (Robinson et al., 2009[111]). Though it can be assumed that there might be some epigenetic alterations for these kind of regulatory changes in gene related to CNS, we couldn't conclude about this because of the lack of study. All these epigenetic modifications with their resulting neuronal diseases are illustrated in Figure 8(Fig. 8).

## 6. Other Developmental Disorders Affected by Cd

Cd is reported to cause various developmental disorders in the liver, kidneys, lungs and forelimbs. Cd interferes with cell differentiation and muscle generation of the developing fetus. Alteration of the placental function was also reported (Stasenko et al., 2010[130]).

The development of the skeletal muscles, cartilage, tendons, endothelial cells and dermis of the embryo relies on the formation and differentiation of somitic cells. *In vitro* exposure to a relatively high dosage of Cd (NO_3_)_2_ resulted in abnormal somitic cell formation (Yamamoto et al., 2012[157]). Cd exposure also alters the methylation pattern of angiogenesis- and cellular damage-regulating genes, which could hamper normal organogenesis (Mohanty et al., 2015[93]). Other organogenesis-related abnormalities, such as forelimb ectrodactyly (Wang Z et al., 2012[151]), irregularities in liver architecture (Abd El-Aziz et al., 2018[2]) and mammary gland formation (Parodi et al., 2017[105]) in rodents, ventral body wall defects, and omphalocele in chick embryos (T. Doi et al., 2011[30]), have been reported after exposure to Cd.

Accumulation of Cd in the fetal kidney causes structural alterations and immature tubular functions. Chronic CdCl_2_ treatment of pregnant SD rats (8.8 mg/kg BW) resulted in degenerated glomeruli, extended urinary space, and increased inflammatory cellular infiltration in offspring (Abd El-Aziz et al., 2018[2]). Previous reports suggested that Cd-induced oxidative stress is responsible for all those alterations (Jacobo-Estrada et al., 2017[59]).

Congenital heart diseases (CHDs) resulting in abnormal cardiac architecture in neonates may occur due to interactions between genetic and environmental factors such as heavy metals (Pb, Cd, As, etc.) (Jin et al., 2016[64]). A recent mouse model study revealed that *in utero* Cd exposure contributes to increased heart weight at birth and hypertension responsiveness in adults. The study also revealed an elevated Na concentration and a lower selenium level in developing fetal blood after Cd exposure, increasing the risk of coronary heart diseases, such as ventricular hypertrophy (Hudson et al., 2019[52]). A particular rodent study revealed severe endothelial dysfunction and adult-age development of ventricular hypertrophy in response to *in utero* exposure to Cd (Ronco et al., 2011[115]). An association between maternal Cd exposure and CHD occurrence was found in a population cohort in which the highest Cd concentration (≥ 25.85 ng/g) in mothers' hair was associated with increased risks of CHDs, such as ventricular septal defects and right and left ventricular outflow tract obstructions (Jin et al., 2016[64]).

Fetal growth restriction is a complication in Cd-exposed fetuses characterized by loss of fetal weight and height, and loss of placental function is suggested to be an underlying cause (Wang H et al., 2016[146]). A particular cohort suggested that placental accumulation of Cd disrupts the transportation of different micronutrients such as Zn (Kippler et al., 2010[71]). According to a separate cohort study, maternal Cd exposure at the middle gestational stage reportedly elevated the risk of SGA in neonates characterized by low-height infants. A higher maternal serum Cd level (≥ 1.06 μg/L) was detected in 10.6% of SGA infants, whereas 7.5% of infants in the lower-serum Cd-exposed group (˂ 1.06 μg/L) were SGA (Wang H, Liu, et al., 2016[146]). Loss of fetal weight can be mediated by the downregulation of glucose transporters (GLUTs), as this gene has been found to be downregulated in the placenta of Cd-treated mice (Xu et al., 2016[156]). The possible mechanism of placental damage may involve cellular apoptosis from the increased endoplasmic reticulum, a decrease in the internal space between maternal and fetal blood vessels, and decreased nutrient transport ability (Wang Z et al., 2012[151]).

Zn is one of the most important nutrients for fetal development. To properly maintain the function of the brain, this element is inevitable (Mimouna et al., 2018[91]). Zn deficiency is considered to be responsible for the abnormalities in various enzyme function and gene expression (Chemek et al., 2015[17]), epigenetic alteration in fetus (Kurita et al., 2013[74]), delayed growth and development of the newborn (Fischer Walker et al., 2009[43]) as well as impairment in cognitive development (Mimouna et al., 2018[91]). It was observed that the homeostasis of Zn in the mammary gland was interrupted due to Cd exposure (Espart et al., 2018[33]). Owing to the presence of Cd in the mammary gland, incompatibility among the Zn transporters was detected. Cd retention in that gland was found to involve the Zn importers ZIP3, ZIP4 and ZIP8, which were upregulated, whereas the Zn exporters ZnT2 and ZnT4 were downregulated. This type of incompatibility in those ZIP protein families has also been observed in the intestines of newborn rats, where Cd is attached to MTs on PNDs. This might create hepatotoxicity and nephrotoxicity when transferred to the liver and kidney (Chemek et al., 2015[17]). Besides, Cd exposure during pregnancy increases the synthesis of MT. This toxic metal then binds with this chelator by displacing Zn from Zn-MT, which hampers the proper distribution of Zn from the mother to the fetus. This kind of modification imposes a threat to both the prenatal and postnatal health of the newborn (Artells et al., 2013[10]; Espart et al., 2018[33]). 

## 7. Summary and Conclusion

In our study, we described the overall journey of Cd from the dietary source of the mother to the offspring successively. The different sources of Cd and their routes of exposure, the mechanism of Cd exposure from the mother to the fetus via fetal circulation and breastmilk, the adverse effects of Cd on the neurodevelopmental process of the fetus in different periods and the epigenetic modifications responsible for those effects are elucidated here. 

Gestational Cd exposure is the root cause of a wide range of disorders in newborns. Owing to this metal's extensive distribution and ability to cross the placental barrier, chronic exposure of the maternal body can impair fetal neurodevelopment and cause irreversible epigenetic modifications. Maternal Cd exposure during the prenatal and perinatal stages has been shown to impair fetal neurodevelopment and induce several neuronal disorders with behavioral and cognitive abnormalities. Different animal and human cohort studies have reported that Cd interferes with the fetal epigenome and gives rise to heritable neuronal diseases. This metal also targets the placenta and blocks the nutrient passage necessary for neural tube closure. In addition, neurogenesis, axonogenesis and neurite formation were shown to be affected in different study models after *in utero* Cd exposure. These complications, together with organogenesis and other congenital disorders, pose a threat to the exposure of newborns to Cd. We also mentioned studies that have shown the long-term effect of Cd exposure which leads to neurodevelopmental delays, reduced head circumference, and impaired skills in children, with some findings suggesting sex-specific vulnerabilities. Research indicates that Cd accumulates more in newborn brains than in adults, contributing to neurobehavioral impairments, learning difficulties, and dyslexia. These effects persist into later life, highlighting Cd's lasting impact on neurological health (Gustin et al., 2018[49]; Kim et al., 2013[68]; Kippler, Tofail, Gardner, et al., 2012[72]; Kippler, Tofail, Hamadani, et al., 2012[73].

Above all, we can conclude that preventing Cd exposure during pregnancy is crucial for safeguarding fetal brain development. The primary sources of Cd exposure are food consumption and smoking, with rice and wheat being significant contributors in regions like Asia with Cd-contaminated soil (Faroon et al., 2013[38]; Tofail et al., 2008[136]). Additionally, house dust exposure can contribute to Cd accumulation (Hogervorst et al., 2007[51]). Future efforts should focus on reducing environmental Cd contamination, promoting awareness among pregnant women, and implementing dietary interventions. Furthermore, postnatal education can help mitigate some cognitive deficits caused by prenatal Cd exposure, highlighting the need for early intervention programs for affected children.

## Notes

Sabiha Sultana Preety and Fahim Rejanur Tasin contributed equally as first author.

## Declaration

### Acknowledgement

This work was partially supported by the Grant funded by Research and Innovation Center (RIC), Khulna University under grant number KU/RC-04/2000-44 to Dr. Chanchal Mandal.

### Conflict of interest

The authors declare that they have no conflict of interest.

### Author contributions

CM, SSP and FRT conceived and designed the content. SSP and FRT constructed all the figures and wrote the manuscript. FY, AS and DH contributed to drafting the article and revising it critically. CM made the final approval of the version to be published.

### Using artificial intelligence (AI) 

We have not used any artificial intelligence (AI)-assisted technologies in the production of submitted work.

## Figures and Tables

**Table 1:**
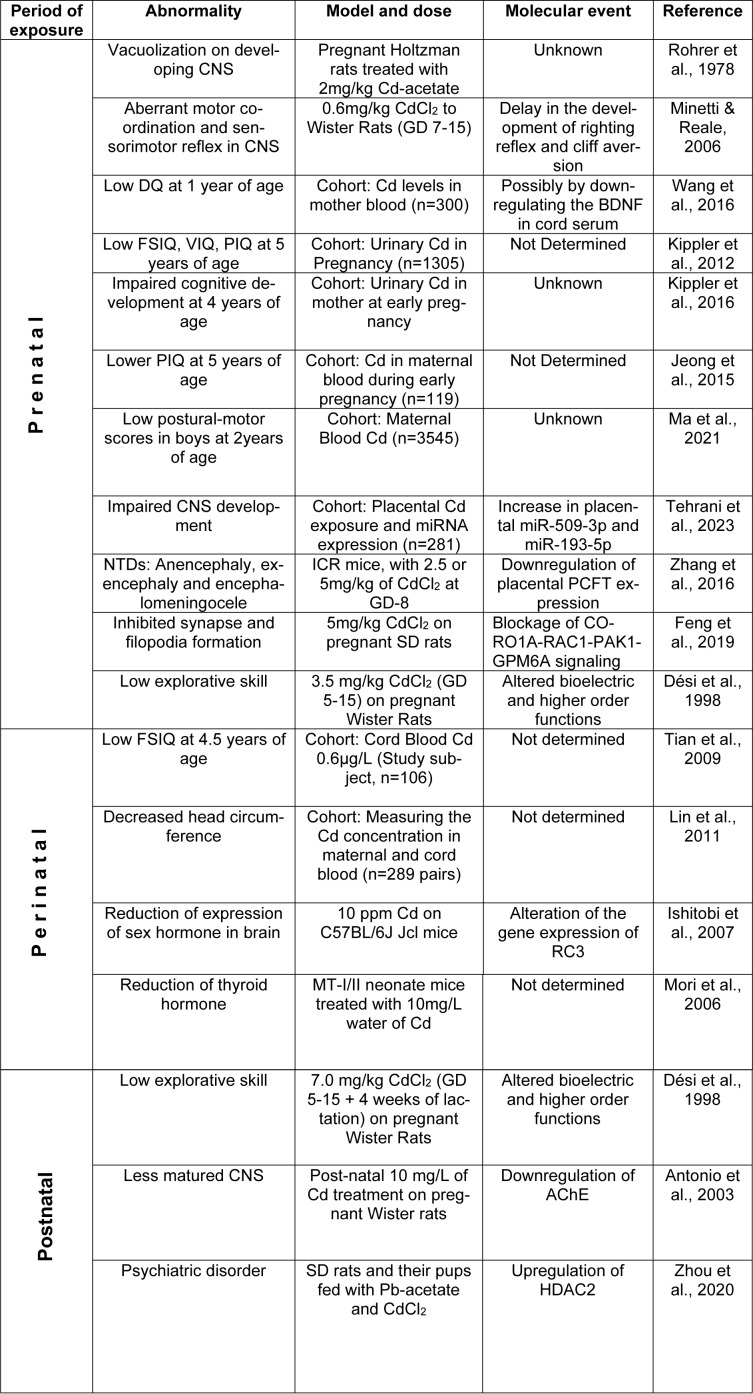
Neuro-related Abnormalities Reported in Recent Experimental Animal or Human Cohort Studies.
